# A Novel Predictive Tool for Poor Anticoagulation Control in Patients on Vitamin K Antagonists in Spain: An Exploratory Study

**DOI:** 10.3390/jcm15134860

**Published:** 2026-06-23

**Authors:** Vivencio Barrios, Manuel Anguita Sánchez, Luis Rodríguez Padial

**Affiliations:** 1 Cardiology Department, Ramon y Cajal University Hospital, University of Alcalá, 28034 Madrid, Spain; 2 Cardiology Department, Reina Sofia University Hospital, University of Córdoba, Maimónides Biomedical Research Institute of Córdoba (IMIBIC), 14004 Córdoba, Spain; 3 Cardiology Department, Cardiovascular Diseases Biomedical Research Networking Centre (CIBERCV), 28029 Madrid, Spain; 4 Cardiology Department, Toledo University Hospital, Servicio de Salud de Castilla-La Mancha (SESCAM), 45007 Toledo, Spain

**Keywords:** predictive, tool, poor, anticoagulation, control, vitamin K antagonists, Spain

## Abstract

**Background**: Given the limitations of available predictive tools for poor anticoagulation control, we aimed to build and validate a novel tool and compare its predictive ability with the SAMe-TT_2_R_2_ score, using the patients from the OBJETIVO 2024 study. **Methods**: The original sample was randomly assigned into a training group (70%, n = 1982) for model development and a validation group (30%, n = 909) for model validation. Stratification of patients was performed based on the presence of diabetes and functional dependence on daily living activities (N with available data = 2891). The model was developed through binary logistic regression, with poor international normalized ratio (INR) control (time in therapeutic range <65% using the Rosendaal method) as a dependent variable. Independent variables included renal insufficiency (glomerular filtration rate < 60 mL/min), chronic obstructive pulmonary disease, diabetes, active smoker, alcohol abuse, previous ablation, hemoglobin level, HbA1c, functional dependence in daily living activities, and number of treatments received in the last 6 months. **Results**: The receiver operating characteristic area under the curve (ROC AUC) was 0.579. The optimal cut-off point was 0.474 (sensitivity: 47.5%; specificity: 65.0%). Overall quality of the model for the training and validation groups was 0.49 and 0.55, respectively. The mean SAMe-TT_2_R_2_ in patients from the OBJETIVO 2024 study was 2.3. The ROC AUC for the SAMe-TT_2_R_2_ tool was 0.530. Overall quality of SAMe-TT_2_R_2_ for the present population was 0.51. **Conclusions**: None of the models presently tested reached the minimum threshold considered acceptable for discriminative ability. To date, utility of different models to predict poor anticoagulation control seems far from optimal in clinical practice.

## 1. Introduction

Atrial fibrillation (AF) is the most frequent cardiac arrhythmia, affecting 1–4% of the population [1]. In Spain, the prevalence of AF is 4.4% of the population aged 40 and over [2]. For decades, vitamin K antagonists (VKAs) have been the only oral anticoagulation therapy available to prevent strokes and systemic embolisms in individuals with AF [3,4]. Nevertheless, studies have revealed that 40–50% of patients with non-valvular atrial fibrillation (NVAF) on VKAs have suboptimal anticoagulation control in Spain [5]. Diverse parameters have been proposed to determine anticoagulation quality. Among them, the time in therapeutic range (TTR) represents the most widely employed [5]. However, TTR is influenced by several factors such as genetics, race, concomitant medications, and diet. In 2013, Apostolakis et al. proposed the SAMe-TT_2_R_2_ score as a clinical prediction model able to estimate the quality of anticoagulation control in patients receiving warfarin [6]. The score integrates a set of sociodemographic and clinical factors, including sex, age, medical history (comorbidities), medication interactions, tobacco use, and race. Diverse studies have validated the SAMe-TT_2_R_2_ score [7,8,9], including in Spanish patients [10,11]. Given the limitations in available predictive tools for poor anticoagulation control, we aimed to develop and validate a novel tool and compare its predictive ability with the SAMe-TT_2_R_2_ score, using the cohort of patients from the OBJETIVO 2024 study (N = 2901) [12,13].

## 2. Materials and Methods

### 2.1. Design of the Study and Patients

The OBJETIVO 2024 was a multicentre, retrospective, and observational study conducted in primary care centers and Cardiology departments of hospitals across Spain [12,13]. Inclusion criteria were: patients over 18 years of age; diagnosed with AF; treated with VKAs for at least 6 months prior to study onset; and with at least 4 international normalized ratio (INR) determinations in the 6 months prior to study onset. Exclusion criteria were: patients with moderate or severe rheumatic mitral stenosis, those with mechanical heart valve prostheses or antiphospholipid syndrome, and patients who were hospitalized at the time of study onset [12,13].

### 2.2. Model Development and SAMe-TT_2_R_2_ Validation

The patient flowchart is shown in Supplementary Figure S1. The original OBJETIVO 2024 study sample was randomly divided into a training group (70%, n = 1982) for model development and a validation group (30%, n = 909) for model validation. Patients were stratified according to the presence of diabetes and functional dependence in daily living activities (N = 2891, with available data, Supplementary Table S1). There were no statistically significant differences between groups in diabetes or functional dependence, indicating the homogeneous distribution of the sample (Supplementary Table S2). Using the training cohort, the tool was developed through binary logistic regression, with poor INR control (TTR < 65% by the Rosendaal method) as the dependent variable. Independent variables included presence of renal insufficiency (glomerular filtration rate < 60 mL/min), chronic obstructive pulmonary disease (COPD), diabetes, active smoker, alcohol abuse, previous ablation, hemoglobin level, HbA1c, functional dependence in daily living activities, and number of treatments received in the last 6 months. Variables were selected from those available in the OBJETIVO 2024 study, based on their inclusion in previous predictive models. To select the best model, potential sub-models were built by combining candidate independent variables and subsequently evaluated using Mallow’s Cp (Supplementary Table S3). The best fitting model was identified as the model with the lowest Mallow’s Cp value (−14.212). Variables in the best model included renal insufficiency, COPD, diabetes, active smoker, alcohol abuse, functional dependence in daily living activities, and number of treatments received in the last 6 months. A total of 1846 patients had data available for the described variables. Poor TTR control was associated with COPD (odds ratio, OR: 1.335), diabetes (OR: 1.248), smoking (OR: 1.481), alcohol abuse (OR: 1.887), and receiving one treatment (OR: 2.174; Supplementary Table S4). Renal insufficiency and functional dependence were close to statistical significance. The formula of the best model is shown in Supplementary Figure S2. Acronyms, definitions, and scores of the SAMe-TT_2_R_2_ model are depicted in Supplementary Table S5. All analyses were carried out using SPSS software, version 30.0. No missing data imputation was performed. Assumptions of missing variables for the SAMe-TT_2_R_2_ score are shown in Supplementary Table S6. The manuscript has been prepared following TRIPOD recommendations [14].

### 2.3. Ethical Approval

The study received approval from the Ethics Committee of Hospital Clínico San Carlos (Madrid, Spain; code 23/011-E; approved on 31 January 2023), which waived the requirement for informed consent because the data were anonymous. The study adhered to the principles of the Declaration of Helsinki.

## 3. Results

### 3.1. Developed Model

**Figure 1:**
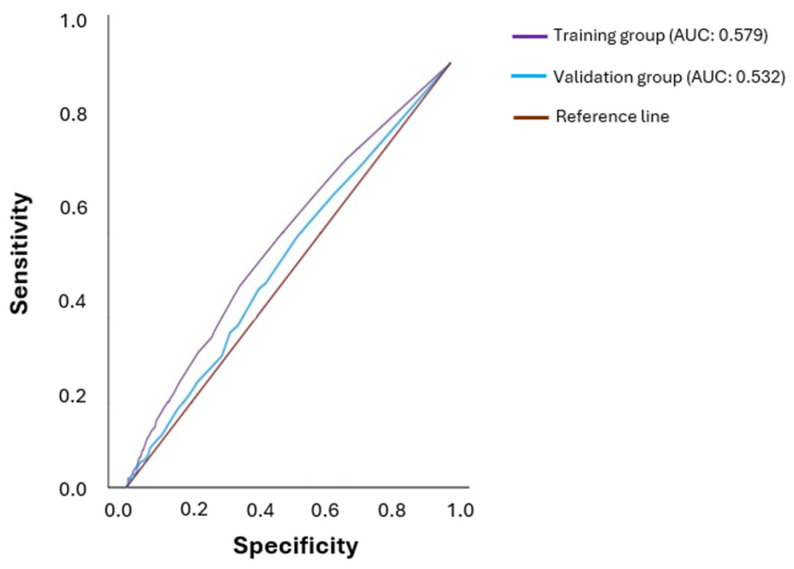
Receiver operating characteristic curves for the training and validation groups. AUC, area under the curve.

The model showed good calibration according to the Hosmer–Lemeshow test (chi-square = 3.986; *p* = 0.679), with no significant differences between predicted and observed values. The receiver operating characteristic area under the curve (ROC AUC) was 0.579 (95% confidence interval, 95% CI: 0.553–0.605; *p* < 0.001; Figure 1), indicating poor discriminative performance [15].

The optimal cut-off point to optimize specificity and sensitivity was 0.474 (yielding a sensitivity of 47.5% and a specificity of 65.0%; Table 1). Observed and predicted cases of INR control using 0.474 as a cut-off value are shown in Supplementary Table S7.

**Figure 2:**
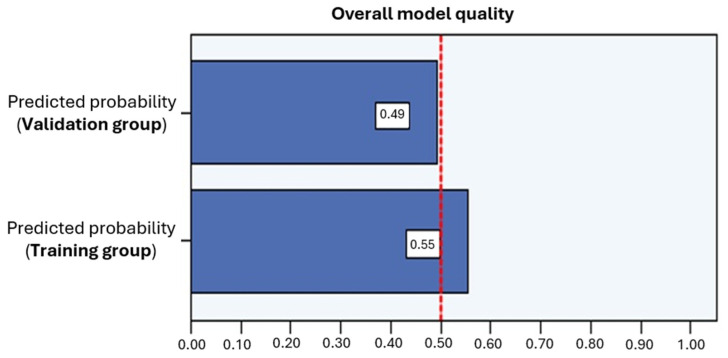
Lower bound of the 95% confidence interval for the receiver operating characteristic area under the curve in the training and validation groups. Values > 0.5 indicate better-than-chance discriminative ability, whereas values ≤ 0.5 indicate no discrimination.

Neither sensitivity nor specificity reached 70%, suggesting that the best model did not adequately discriminate between patients with good or poor INR control. In addition, the Spearman coefficient R_b_ between observed and predicted values showed a weak correlation (coefficient = −0.087; *p* = 0.012), indicating an inverse relationship. Using the formula derived from the best model, predicted values were then calculated for patients in the validation group. The ROC AUC in this group was 0.532 (95% CI: 0.493–0.571; Figure 1), and the Spearman coefficient Rb also showed the same weak correlation (coefficient = −0.087; *p* = 0.012). Significant differences were found in ROC AUC between training and validation groups (*p* = 0.048). Figure 2 depicts the lower bound of the 95% CI for the ROC AUC in the training (0.49) and validation (0.55) groups, as a conservative estimate of model performance.

### 3.2. SAMe-TT_2_R_2_ Validation

**Figure 3:**
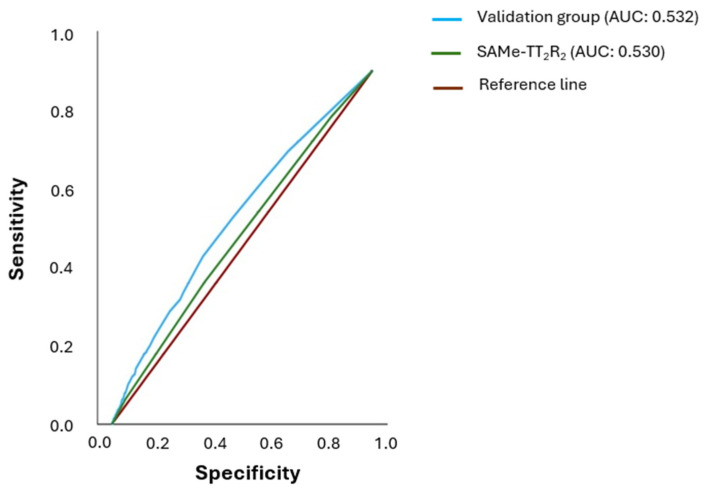
Receiver operating characteristic curves for the validation and SAMe-TT_2_R_2_ groups. AUC, area under the curve.

The mean SAMe-TT_2_R_2_ in patients from OBJETIVO 2024 was 2.3 (standard deviation: 0.8). The ROC AUC for the SAMe-TT_2_R_2_ tool was 0.530 (95% CI: 0.508–0.553; *p* = 0.007; Figure 3); thus, the model showed poor discrimination.

The optimal cut-off point was three (yielding a sensitivity of 40.3% and a specificity of 64.4%; Table 2). Observed and predicted cases of INR control using three as the cut-off value in the SAMe-TT_2_R_2_ validation group are shown in Supplementary Table S8.

**Figure 4:**
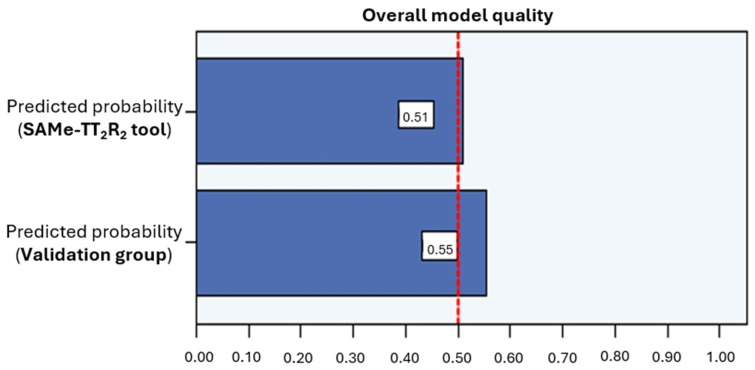
Lower bound of the 95% confidence interval for the receiver operating characteristic area under the curve. Overall quality of the model in the validation and SAMe-TT_2_R_2_ groups. Values > 0.5 indicate better-than-chance discriminative ability, whereas values ≤ 0.5 indicate no discrimination.

Significant differences were found in ROC AUC between the validation group and the SAMe-TT_2_R_2_ tool (*p* = 0.005). Figure 4 depicts the lower bound of the 95% CI for the ROC AUC in the SAMe-TT_2_R_2_ (0.51) and validation (0.55) groups, as a conservative estimate of model performance.

## 4. Discussion

There remains a critical need for novel predictive tools to identify patients at higher risk of poor INR control. The SAMe-TT_2_R_2_ score has been previously validated in diverse studies from Spain [16,17]. In the FANTASIIA registry, involving data from 1470 patients with AF and one-year follow-up on VKAs, a SAMe-TT_2_R_2_ score > 2 was associated with a specificity of >90% for predicting a TTR < 70% [16]. Thus, the FANTASIIA registry identified aSAMe-TT_2_R_2_ > 2 as an indicator of poor control. By contrast, other studies have reported a modest predictive value for the SAMe-TT_2_R_2_ score [17,18]. In the observational, cross-sectional, retrospective and nationwide multicenter PAULA study, with data from 1524 patients with NVAF receiving VKAs in primary care settings, the mean TTR progressively decreased as the SAMe-TT_2_R_2_ score increased from 0 to 4 points [17]. Nevertheless, the ROC curve showed a low capability for discriminating between good and poor anticoagulation control, with an AUC of 0.562. In another study by Andreu-Cayuelas et al. [18], a retrospective analysis of 108 patients with NVAF discharged after heart failure and treated with VKA therapy showed similar mean SAMe-TT_2_R_2_ scores in patients with TTR ≥ 65% (1.9) and those with TTR < 65% (2.1). Furthermore, no differences were reported in the proportion of patients with a SAMe-TT_2_R_2_ score ≥ 2 between those with TTR ≥ 65% (69%) and those with TTR < 65% (75%). In addition, a study evaluating data from PAULA and FANTASIIA developed the DAFNE score (cardiovascular disease, concomitant treatment with amiodarone, female sex, dietary transgression, and taking ≥7 pills daily) and found that higher scores were associated with greater probability of poor INR control [19]. In our present study, variables that could better discriminate inadequate anticoagulation control included renal insufficiency, COPD, diabetes, active smoking, alcohol abuse, previous ablation, hemoglobin level, HbA1c, functional dependence, and treatments received in the last 6 months. However, the developed model showed only modest predictive ability. Similarly, the SAMe-TT_2_R_2_ score demonstrated limited utility in assessing poor anticoagulation control in patients from OBJETIVO 2024. Given the overall poor performance of both models, the results should be interpreted cautiously.

The major limitation of our study was the post hoc nature of the analysis, which was not originally planned in the OBJETIVO 2024 study. As a consequence, the study design was not ideally suited to develop a predictive tool for poor INR control, raising concerns about variable selection, model stability, and generalizability. In addition, as the study was retrospective, only variables collected from clinical practice were available. For this reason, information on missing variables, such as polypharmacy, might have enhanced the predictive value of the tool by capturing drug interactions and adherence-related variability, thereby accounting for a greater proportion of the factors influencing INR control. Furthermore, some variables required for SAMe-TT_2_R_2_ calculation were not available (coronary artery disease, previous stroke, duration of active smoking or abstinence, and race); therefore, certain assumptions had to be made. In addition, the risk of model overfitting cannot be excluded, especially considering the number of variables evaluated in relation to the sample size. Moreover, residual confounding due to unmeasured or inadequately controlled factors may have influenced the observed associations; therefore, the results should be interpreted with caution.

Besides these limitations, in our opinion, the potential value of our model lies in its exploratory nature, as the variables identified may be applied in future studies and in models based on a larger cohort of patients and incorporating variables currently unmeasured.

Overall, our findings are consistent with the relatively poor performance of predictive models reported in previous studies [17,18]. One possible explanation is that achieving accurate prediction of inadequate INR control in patients treated with VKAs may be inherently challenging. This difficulty likely reflects the multifactorial nature of anticoagulation control, where numerous clinical, behavioral, and biological determinants interact simultaneously [20,21]. In addition, genetic determinants, including polymorphisms in CYP2C9 and VKORC1, have been shown to modulate sensitivity to or resistance against VKAs [22]. However, as this study was based on clinical practice, genetic data were not available.

## 5. Conclusions

None of the models tested reached the minimum threshold considered acceptable for discriminative ability. To date, the utility of different models to predict poor anticoagulation control seems far from optimal in clinical practice. Further efforts are warranted to develop tools capable of better identifying patients at greater risk of poor anticoagulation control.

## Figures and Tables

**Table 1:** Values obtained from receiver operating characteristic curves in the training and validation groups.

Value (95% CI)	Training Group (Cut-Off 0.474)	Validation Group (Cut-Off 0.474)
Sensitivity	47.5% (44.2–50.8%)	48.1% (43.3–53.0%)
Specificity	65.0% (61.9–68.0%)	57.0% (52.4–61.5%)
Positive Predictive Value	55.9% (52.3–59.4%)	50.4% (45.4–55.4%)
Negative Predictive Value	57.0% (54.1–59.9%)	54.8% (50.2–59.3%)
Overall value (Efficiency)	56.6% (54.3–58.8%)	52.8% (49.4–56.1%)

CI, confidence interval.

**Table 2:** Values obtained from receiver operating characteristic curves with SAMe-TT_2_R_2_ score.

Value (95% CI)	SAMe-TT_2_R_2_ (Cut-off 3)
Sensitivity	40.3% (37.6–43.0%)
Specificity	64.4% (61.8–66.9%)
Positive Predictive Value	51.2% (48.1–54.3%)
Negative Predictive Value	53.7% (51.2–56.1%)
Overall Value (Efficiency)	52.8% (50.8–54.7%)

CI, confidence interval.

## Data Availability

The data supporting the findings of this study are not publicly available; however, they can be made available from the corresponding author upon reasonable request.

## Supplementary Materials

The following supporting information can be downloaded at https://www.mdpi.com/article/10.3390/jcm15134860/s1. Figure S1: Patient flowchart; Table S1: Stratification of patients regarding the presence of diabetes and functional dependence in daily living activities; Table S2: Stratification of patients regarding the presence of diabetes and functional dependence in daily living activities in training and validation groups; Table S3: Best models adjusted according to Mallows’ Cp criterion; Table S4: Best logistic regression model; Figure S2: Formula of the best model; Table S5: Acronyms, definitions, and score in SAMe-TT_2_R_2_ model; Table S6: Assumptions of missing variables for the SAMe-TT_2_R_2_ score; Table S7: Observed and predicted cases of INR control using 0.474 as the cut-off value in training and validation groups; Table S8: Observed and predicted cases of INR control using 3 as the cut-off value in the SAMe-TT_2_R_2_ validation group.

## Abbreviations

The following abbreviations are used in this manuscript: AF, atrial fibrillation; VKAs, vitamin K antagonists; NVAF, non-valvular atrial fibrillation; TTR, time in therapeutic range; INR, international normalized ratio; COPD, chronic obstructive pulmonary disease; ROC AUC, receiver operating characteristic area under the curve.
