# Supplementary material for: A Novel Predictive Tool for Poor Anticoagulation Control in Patients on Vitamin K Antagonists in Spain: An Exploratory Study
Source: J Clin Med. 2026 Jun 23;15(13):4860. doi: 10.3390/jcm15134860 (PMC13362016; doi:10.3390/jcm15134860)
Supplement: Supplementary file 1 [file jcm-15-04860-s001.zip › jcm-4307255-supplementary.pdf]

Supplementary Figure S1. Patient flowchart

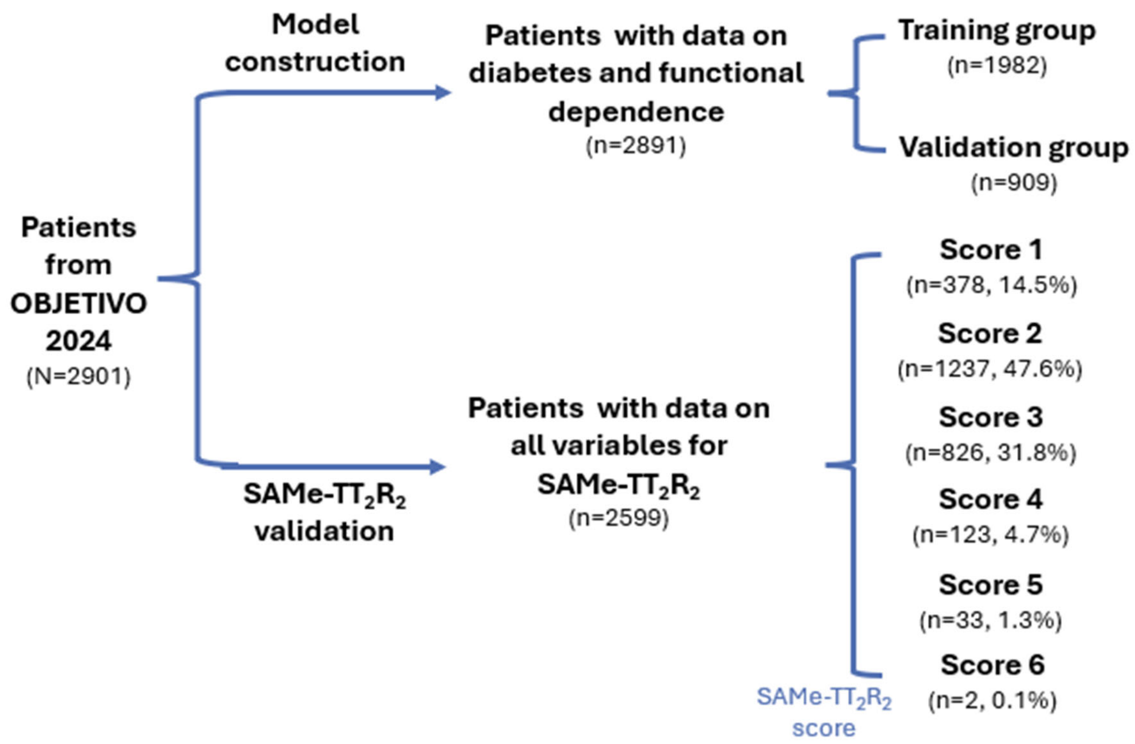

Supplementary Table S1. Stratification of patients regarding the presence of diabetes and functional dependence in daily living activities

	**Patients**<br>n (%)
Patients with incomplete data	10 (0.3)
Patients with complete data	2891 (99.7)
Patients with no diabetes and no functional dependence	1423 (49.2)
Patients with no diabetes and functional dependence	518 (17.9)
Patients with diabetes and no functional dependence	660 (22.8)
Patients with diabetes and functional dependence	290 (10.0)

Supplementary Table S2. Stratification of patients regarding the presence of diabetes and functional dependence in daily living activities in training and validation groups

	**Training group** n (%)	**Validation group** n (%)	**p-value**
Diabetes mellitus			0.495
No	1339 (67.6)	602 (66.2)	
Yes	643 (32.4)	307 (33.8)	
Functional dependence			0.964
No	1429 (72.1)	654 (71.9)	2083 (72.1)
Yes	1982 (100.0)	909 (100.0)	2891 (100.0)

Supplementary Table S3. Best models adjusted according to Mallows' Cp criterion

Variable	Variable labeling in multivariable models	Value labels
cMetRoos	Control defined according to the Rosendaal method	0: Good control / 1: Poor control
RI	Renal insufficiency (glomerular filtration rate < 60 ml/min)	(0: No / 1: Yes)
COPD	Chronic obstructive pulmonary disease	(0: No / 1: Yes)
DM	Diabetes mellitus	(0: No / 1: Yes)
SMOKER	Active smoker	(0: No / 1: Yes)
ALCOHOL	Alcohol abuse	(0: No / 1: Yes)
PrevAbla	Previous ablation	(0: No / 1: Yes)
Hb	Hemoglobin level (number)	-
HbA1c	HbA1c (number)	-
FuncDep	Does the patient exhibit functional dependence in performing activities of daily living, either basic or instrumental?	(0: No / 1: Yes)
NTx	Number of treatments received in the last 6 months (categorical)	(0: 1 / 1: >1)

Subsets of 1 variable (Cp criterion)

	Cp	AUC	Se	Sp	-2 LL
Hb	-1.110	0.540	38.8%	66.9%	2461.6
SMOKER	-0.726	0.516	8.6%	94.7%	2643.0
ALCOHOL	-0.171	0.511	4.2%	98.0%	2607.6
COPD	0.971	0.519	15.2%	88.5%	2714.8
DM	1.311	0.525	35.0%	70.0%	2739.9
NTx	2.419	0.507	3.1%	98.3%	2740.9
PrevAbla	2.609	0.509	0.0%	100.0%	2620.7
RI	2.681	0.522	36.2%	68.2%	2716.6
FuncDep	3.607	0.519	29.8%	73.9%	2742.2
HbA1c	6.326	0.518	9.2%	92.5%	1282.9

Cp, Mallows' Cp; AUC, area under the curve; Se, sensitivity; Sp, specificity; -2 LL, −2 log likelihood; Hb, hemoglobin level; COPD, chronic obstructive pulmonary disease; DM, diabetes mellitus; PrevAbla, previous ablation; FuncDep, functional dependence; NTx, Number of treatments

Subsets of 2 variables (Cp criterion)

	Cp	AUC	Se	Sp	-2 LL
ALCOHOL. Hb	-8.326	0.553	33.8%	73.9%	2343.7
COPD. Hb	-4.367	0.546	38.8%	68.7%	2438.3
COPD. SMOKER	-4.104	0.535	21.1%	85.9%	2614.2
COPD. ALCOHOL	-4.078	0.530	18.3%	87.5%	2578.3
DM. ALCOHOL	-3.746	0.533	37.6%	68.4%	2602.0

DM. SMOKER	-3.653	0.537	40.0%	66.7%	2638.1
SMOKER. FuncDep	-3.588	0.537	37.4%	69.3%	2638.2
SMOKER. NTx	-3.373	0.523	11.5%	93.0%	2638.3
SMOKER. Hb	-3.360	0.555	37.5%	71.4%	2380.7
PrevAbla. Hb	-2.761	0.545	44.4%	63.1%	2351.2

Cp, Mallows' Cp; AUC, area under the curve; Se, sensitivity; Sp, specificity; -2 LL, −2 log likelihood; Hb, hemoglobin level; COPD, chronic obstructive pulmonary disease; DM, diabetes mellitus; PrevAbla, previous ablation; FuncDep, functional dependence; NTx, Number of treatments

Subsets of 3 variables (Cp criterion)

	Cp	AUC	Se	Sp	-2 LL
COPD. ALCOHOL. Hb	-11.182	0.559	36.3%	73.8%	2320.8
DM. ALCOHOL. Hb	-8.747	0.556	36.1%	70.9%	2341.2
ALCOHOL. Hb. NTx	-8.707	0.558	33.8%	75.0%	2341.3
ALCOHOL. Hb. FuncDep	-8.496	0.563	36.3%	72.7%	2341.5
SMOKER. ALCOHOL. Hb	-8.093	0.560	35.0%	73.6%	2319.6
ALCOHOL. PrevAbla. Hb	-7.963	0.557	40.1%	68.9%	2252.1
RI. ALCOHOL. Hb	-7.557	0.554	37.3%	72.2%	2324.6
DM. SMOKER. NTx	-6.752	0.546	42.6%	65.6%	2632.9
COPD. DM. ALCOHOL	-6.681	0.545	46.1%	61.6%	2573.7
DM. ALCOHOL. NTx	-6.606	0.542	40.2%	67.3%	2597.1

Cp, Mallows' Cp; AUC, area under the curve; Se, sensitivity; Sp, specificity; -2 LL, −2 log likelihood; Hb, hemoglobin level; COPD, chronic obstructive pulmonary disease; DM, diabetes mellitus; PrevAbla, previous ablation; FuncDep, functional dependence; NTx, Number of treatments

Subsets of 4 variables (Cp criterion)

	Cp	AUC	Se	Sp	-2 LL
COPD. ALCOHOL. Hb. NTx	-11.607	0.565	37.2%	73.8%	2318.3
COPD. ALCOHOL. PrevAbla. Hb	-11.103	0.563	40.6%	69.7%	2231.6
COPD. DM. ALCOHOL. Hb	-11.072	0.561	37.8%	70.4%	2318.9
COPD. ALCOHOL. Hb. FuncDep	-11.021	0.567	38.6%	70.2%	2318.9
COPD. SMOKER. ALCOHOL. Hb	-10.294	0.567	37.2%	73.9%	2297.4
RI. COPD. ALCOHOL. Hb	-9.998	0.560	38.4%	69.7%	2307.6
RI. COPD. SMOKER. NTx	-9.305	0.567	23.9%	84.7%	2594.1
DM. SMOKER. FuncDep. NTx	-9.280	0.558	21.6%	84.7%	2628.4
DM. ALCOHOL. Hb. NTx	-9.266	0.562	35.5%	72.1%	2338.7
RI. DM. SMOKER. NTx	-9.123	0.562	24.8%	83.2%	2608.2

Cp, Mallows' Cp; AUC, area under the curve; Se, sensitivity; Sp, specificity; -2 LL, −2 log likelihood; Hb, hemoglobin level; COPD, chronic obstructive pulmonary disease; DM, diabetes mellitus; PrevAbla, previous ablation; FuncDep, functional dependence; NTx, Number of treatments

Subsets of 5 variables (Cp criterion)

	Cp	AUC	Se	Sp	-2 LL
RI. COPD. DM. ALCOHOL. NTx	-11.923	0.566	32.2%	77.0%	2552.0
RI. COPD. DM. SMOKER. NTx	-11.802	0.573	35.0%	75.9%	2589.6
COPD. DM. ALCOHOL. Hb. NTx	-11.620	0.568	38.1%	71.2%	2316.3
COPD. ALCOHOL. PrevAbla. Hb. FuncDep	-11.522	0.570	41.8%	67.9%	2229.2
COPD. ALCOHOL. Hb. FuncDep. NTx	-11.349	0.572	38.4%	70.5%	2316.6
RI. COPD. ALCOHOL. Hb. NTx	-11.270	0.568	39.2%	70.0%	2304.3
RI. DM. SMOKER. ALCOHOL. NTx	-11.238	0.562	27.1%	81.1%	2544.3
DM. SMOKER. ALCOHOL. FuncDep. NTx	-11.109	0.559	23.7%	82.8%	2564.7
COPD. SMOKER. ALCOHOL. Hb. NTx	-10.904	0.573	37.6%	74.0%	2294.7
COPD. DM. ALCOHOL. PrevAbla. Hb	-10.873	0.563	40.7%	67.4%	2229.8

Cp, Mallows' Cp; AUC, area under the curve; Se, sensitivity; Sp, specificity; -2 LL, −2 log likelihood; Hb, hemoglobin level; COPD, chronic obstructive pulmonary disease; DM, diabetes mellitus; PrevAbla, previous ablation; FuncDep, functional dependence; NTx, Number of treatments

Subsets of 6 variables (Cp criterion)

	Cp	AUC	Se	Sp	-2 LL
RI. COPD. DM. SMOKER. ALCOHOL. NTx	-13.124	0.573	32.8%	77.4%	2526.4
RI. COPD. DM. SMOKER. FuncDep. NTx	-12.895	0.578	42.7%	68.8%	2586.5
RI. DM. SMOKER. ALCOHOL. FuncDep. NTx	-12.794	0.569	40.2%	70.5%	2540.7
RI. COPD. DM. ALCOHOL. FuncDep. NTx	-12.650	0.572	39.6%	70.3%	2549.2
COPD. DM. SMOKER. ALCOHOL. FuncDep. NTx	-11.712	0.570	33.4%	75.9%	2538.7
RI. COPD. DM. ALCOHOL. Hb. NTx	-11.527	0.571	41.3%	70.0%	2302.0
RI. COPD. SMOKER. ALCOHOL. FuncDep. NTx	-11.468	0.575	37.4%	74.6%	2528.1
COPD. SMOKER. ALCOHOL. PrevAbla. Hb. FuncDep	-11.327	0.575	43.3%	67.4%	2207.8
COPD. DM. ALCOHOL. Hb. FuncDep. NTx	-11.297	0.572	39.1%	68.4%	2314.6
COPD. ALCOHOL. PrevAbla. Hb. FuncDep. NTx	-11.217	0.575	42.2%	68.1%	2227.5

Cp, Mallows' Cp; AUC, area under the curve; Se, sensitivity; Sp, specificity; -2 LL, −2 log likelihood; Hb, hemoglobin level; COPD, chronic obstructive pulmonary disease; DM, diabetes mellitus; PrevAbla, previous ablation; FuncDep, functional dependence; NTx, Number of treatments

Subsets of 7 variables (Cp criterion)

	Cp	AUC	Se	Sp	-2 LL
**RI. COPD. DM. SMOKER. ALCOHOL. FuncDep. NTx[a]**	**-14.212**	**0.579**	**44.6%**	**67.2%**	**2523.3**
COPD. SMOKER. ALCOHOL. PrevAbla. Hb. FuncDep. NTx	-11.204	0.579	43.1%	67.9%	2205.9

COPD. DM. ALCOHOL. PrevAbla. Hb. FuncDep. NTx	-10.997	0.574	42.6%	66.1%	2225.7
RI. COPD. DM. ALCOHOL. Hb. FuncDep. NTx	-10.915	0.573	40.0%	68.9%	2300.6
COPD. DM. SMOKER. ALCOHOL. PrevAbla. Hb. FuncDep	-10.893	0.573	42.6%	66.2%	2206.2
RI. COPD. DM. SMOKER. ALCOHOL. Hb. NTx	-10.874	0.578	42.0%	71.0%	2279.8
COPD. DM. SMOKER. ALCOHOL. PrevAbla. FuncDep. NTx	-10.631	0.569	32.3%	76.1%	2442.2
COPD. DM. SMOKER. ALCOHOL. Hb. FuncDep. NTx	-10.595	0.578	40.1%	69.6%	2291.0
COPD. DM. SMOKER. ALCOHOL. PrevAbla. Hb. NTx	-10.534	0.573	41.8%	69.5%	2206.6
RI. COPD. SMOKER. ALCOHOL. Hb. FuncDep. NTx	-10.292	0.579	40.2%	70.3%	2280.4

a. Best model

Cp, Mallows' Cp; AUC, area under the curve; Se, sensitivity; Sp, specificity; -2 LL, −2 log likelihood; Hb, hemoglobin level; COPD, chronic obstructive pulmonary disease; DM, diabetes mellitus; PrevAbla, previous ablation; FuncDep, functional dependence; NTx, Number of treatments

Subsets of 8 variables (Cp criterion)

	Cp	AUC	Se	Sp	-2 LL
RI. COPD. DM. SMOKER. ALCOHOL. PrevAbla. FuncDep. NTx	-11.021	0.575	43.1%	67.5%	2430.4
COPD. DM. SMOKER. ALCOHOL. PrevAbla. Hb. FuncDep. NTx	-10.894	0.577	43.3%	66.3%	2204.2
RI. COPD. DM. SMOKER. ALCOHOL. Hb. FuncDep. NTx	-10.381	0.579	41.2%	68.4%	2278.3
RI. COPD. SMOKER. ALCOHOL. PrevAbla. Hb. FuncDep. NTx	-9.724	0.579	44.1%	67.7%	2195.9
RI. COPD. DM. ALCOHOL. PrevAbla. Hb. FuncDep. NTx	-9.705	0.574	42.9%	65.7%	2214.0
RI. COPD. DM. SMOKER. ALCOHOL. PrevAbla. Hb. NTx	-9.432	0.576	44.2%	67.9%	2196.2
RI. COPD. DM. SMOKER. ALCOHOL. PrevAbla. Hb. FuncDep	-8.917	0.573	43.6%	65.2%	2196.7
RI. DM. SMOKER. ALCOHOL. PrevAbla. Hb. FuncDep. NTx	-8.184	0.574	43.6%	66.3%	2208.6
RI. COPD. DM. SMOKER. PrevAbla. Hb. FuncDep. NTx	-5.521	0.574	45.0%	64.3%	2247.4
RI. COPD. DM. SMOKER. Hb. HbA1c. FuncDep. NTx	8.243	0.573	33.1%	75.4%	1195.1

Cp, Mallows' Cp; AUC, area under the curve; Se, sensitivity; Sp, specificity; -2 LL, −2 log likelihood; Hb, hemoglobin level; COPD, chronic obstructive pulmonary disease; DM, diabetes mellitus; PrevAbla, previous ablation; FuncDep, functional dependence; NTx, Number of treatments

Subsets of 9 variables (Cp criterion)

	Cp	AUC	Se	Sp	-2 LL
RI. COPD. DM. SMOKER. ALCOHOL. PrevAbla. Hb. FuncDep. NTx	-9.610	0.579	44.2%	65.9%	2194.0
RI. DM. SMOKER. ALCOHOL. PrevAbla. Hb. HbA1c. FuncDep. NTx	9.440	0.573	35.5%	73.4%	1125.8

COPD. DM. SMOKER. ALCOHOL. PrevAbla. Hb. HbA1c. FuncDep. NTx	9.492	0.573	35.9%	74.1%	1119.9
RI. COPD. DM. SMOKER. ALCOHOL. Hb. HbA1c. FuncDep. NTx	9.880	0.571	34.7%	75.1%	1157.3
RI. COPD. SMOKER. ALCOHOL. PrevAbla. Hb. HbA1c. FuncDep. NTx	10.155	0.571	35.6%	72.4%	1118.0
RI. COPD. DM. SMOKER. PrevAbla. Hb. HbA1c. FuncDep. NTx	10.234	0.573	36.8%	74.3%	1148.4
RI. COPD. DM. SMOKER. ALCOHOL. PrevAbla. Hb. HbA1c. NTx	10.649	0.568	36.4%	75.2%	1118.5
RI. COPD. DM. SMOKER. ALCOHOL. PrevAbla. HbA1c. FuncDep. NTx	10.771	0.563	33.1%	74.8%	1134.0
RI. COPD. DM. SMOKER. ALCOHOL. PrevAbla. Hb. HbA1c. FuncDep	11.073	0.566	36.2%	72.4%	1118.9
RI. COPD. DM. ALCOHOL. PrevAbla. Hb. HbA1c. FuncDep. NTx	11.240	0.564	34.6%	73.7%	1131.8

Cp, Mallows' Cp; AUC, area under the curve; Se, sensitivity; Sp, specificity; -2 LL, −2 log likelihood; Hb, hemoglobin level; COPD, chronic obstructive pulmonary disease; DM, diabetes mellitus; PrevAbla, previous ablation; FuncDep, functional dependence; NTx, Number of treatments

Subsets of 10 (all) variables (Cp criterion)

	Cp	AUC	Se	Sp	-2 LL
**RI. COPD. DM. SMOKER. ALCOHOL. PrevAbla. Hb. HbA1c. FuncDep. NTx**	11.000	0.574	37.4%	73.1%	1116.8

Cp, Mallows' Cp; AUC, area under the curve; Se, sensitivity; Sp, specificity; -2 LL, −2 log likelihood; Hb, hemoglobin level; COPD, chronic obstructive pulmonary disease; DM, diabetes mellitus; PrevAbla, previous ablation; FuncDep, functional dependence; NTx, Number of treatments

SUMMARY OF BEST SUBSETS (Cp criterion)

	Cp	AUC	Se	Sp	-2 LL
All variables	**11.000**	**0.574**	**37.4%**	**73.1%**	**1116.8**
**RI. COPD. DM. SMOKER. ALCOHOL. FuncDep. NTx**	**-14.212**	**0.579**	**44.6%**	**67.2%**	**2523.3**
RI. COPD. DM. SMOKER. ALCOHOL. NTx	-13.124	0.573	32.8%	77.4%	2526.4
RI. COPD. DM. SMOKER. FuncDep. NTx	-12.895	0.578	42.7%	68.8%	2586.5
RI. DM. SMOKER. ALCOHOL. FuncDep. NTx	-12.794	0.569	40.2%	70.5%	2540.7
RI. COPD. DM. ALCOHOL. FuncDep. NTx	-12.650	0.572	39.6%	70.3%	2549.2
RI. COPD. DM. ALCOHOL. NTx	-11.923	0.566	32.2%	77.0%	2552.0
RI. COPD. DM. SMOKER. NTx	-11.802	0.573	35.0%	75.9%	2589.6
COPD. DM. SMOKER. ALCOHOL. FuncDep. NTx	-11.712	0.570	33.4%	75.9%	2538.7
COPD. DM. ALCOHOL. Hb. NTx	-11.620	0.568	38.1%	71.2%	2316.3

COPD. ALCOHOL. Hb. NTx	-11.607	0.565	37.2%	73.8%	2318.3

Cp, Mallows' Cp; AUC, area under the curve; Se, sensitivity; Sp, specificity; -2 LL, −2 log likelihood; Hb, hemoglobin level; COPD, chronic obstructive pulmonary disease; DM, diabetes mellitus; PrevAbla, previous ablation; FuncDep, functional dependence; NTx, Number of treatments

Supplementary Table S4. Best logistic regression model

**Best model**	**B**	**Odds ratio (95%CI)**	**p-value**
Renal insufficiency (No / Yes)	0.192	1.212 (0.995-1.476)	0.056
Chronic obstructive pulmonary disease (No / Yes)	0.289	1.335 (1.008-1.768)	0.044
Diabetes mellitus (No / Yes)	0.221	1.248 (1.024-1.521)	0.028
Smoker (No / Yes)	0.393	1.481 (1.013-2.164)	0.042
Alcohol (No / Yes)	0.635	1.887 (1.072-3.323)	0.028
Functional dependence (in daily living activities; No / Yes)	0.187	1.206 (0.980-1.484)	0.077
Number of treatments received in the last 6 months (1 / >1)	-0.776	0.460 (0.242-0.876)	0.018
Constant	0.417	1.518	0.200

Supplementary Figure S2. Formula of the best model

$$\text{Formula} = 1/(1+e^{-(0.417 + 0.192 \times \text{RI} + 0.289 \times \text{COPD} + 0.221 \times \text{DM} + 0.393 \times \text{SMOKE} + 0.635 \times \text{ALCOHOL} + 0.187 \times \text{FunDep} - 0.776 \times \text{NTx})})$$

RI, renal insufficiency; COPD, chronic obstructive pulmonary disease; DM, diabetes mellitus; FuncDep, Functional dependence (in daily living activities); NTx, number of treatments received in the last 6 months

Supplementary Table S5. Acronyms, definitions, and score in SAMe-TT_2R_2 model

**Acronym**	**Definition**	**Score**
S	Sex (female)	1
A	Age (<60 years)	1
Me	Medical history[)]	1
T	Treatment	1
T	Tabacco (during the last 2 years)	2
R	Race (no Caucasic	2

Supplementary Table S6. Assumptions of missing variables for the SAMe-TT_2R_2 score

Original (missing) variable	Assumed variable/s
Coronary artery disease	- Ischemic heart disease<br>- Myocardial infarction
Prior stroke	- Stroke<br>- Transient ischemic attack
Treatments	- Vitamin K antagonists<br>- Antiplatelet agents<br>- Angiotensin-converting enzyme inhibitors<br>- Angiotensin II receptor blockers<br>- Diuretics<br>- Statins<br>- Beta-blockers<br>- Calcium channel blockers<br>- Antiarrhythmics<br>- Amiodarone<br>- Regular use of non-steroidal anti-inflammatory drugs (at least once per week)
Smoker: Smoking duration or cessation time	- Only patients classified as smokers (Yes) were considered for this item
Race	- All patients were considered Caucasian

Supplementary Table S7. Observed and predicted cases of INR control using 0.474 as cut-off value in training and validation groups

			PREDICTED		
			INR control *		
			Good control	Poor control	% correct
**TRAINING GROUP**					
**OBSERVED**	INR control *	Good control	621	335	65.0
		Poor control	468	423	47.5
% global					**56.6**
**VALIDATION GROUP**					
**OBSERVED**	INR control *	Good control	252	190	57.0
		Poor control	208	193	48.1
% global					**52.8**

INR, international normalized ratio

* Good control defined as ≤ 0.474

Supplementary Table S8. Observed and predicted cases of INR control using 3 as cut-off value in the SAMe-TT_2R_2 validation group

			PREDICTED		
			SAMe-TT_2R_2		
			<3	≥3	% correct
**OBSERVED**	INR control	Good control	867	480	64.4
		Poor control	748	504	40.3
% global					**52.8**

INR, international normalized ratio
